# The School Health Index as an Impetus for Change^1^

**Published:** 2004-12-15

**Authors:** Lisa K Staten, Nicolette I Teufel-Shone, Victoria E Steinfelt, Nohemi Ortega, Karen Halverson, Carmen Flores, Michael D Lebowitz

**Affiliations:** Division of Health Promotion Sciences and Southwest Center for Community Health Promotion, Mel and Enid Zuckerman Arizona College of Public Health, University of Arizona; Division of Health Promotion Sciences and Southwest Center for Community Health Promotion, Mel and Enid Zuckerman Arizona College of Public Health, University of Arizona, Tucson, Ariz; Cooperative Extension, College of Agriculture and Life Sciences, University of Arizona, Yuma, Ariz; Cooperative Extension, College of Agriculture and Life Sciences, University of Arizona, Yuma, Ariz; Southeast Arizona Area Health Education Center, Nogales, Ariz; Southeast Arizona Area Health Education Center, Nogales, Ariz; Arizona Prevention Center and Southwest Center for Community Health Promotion, Mel and Enid Zuckerman Arizona College of Public Health, University of Arizona, Tucson, Ariz

## Abstract

**Background:**

The increase in childhood obesity and prevalence of chronic disease risk factors demonstrate the importance of creating healthy school environments. As part of the Border Health Strategic Initiative, the School Health Index was implemented in public schools in two counties along the Arizona, United States-Sonora, Mexico border. Developed in 2000 by the Centers for Disease Control and Prevention, the School Health Index offers a guide to assist schools in evaluating and improving opportunities for physical activity and good nutrition for their students.

**Context:**

Between 2000 and 2003, a total of 13 schools from five school districts in two counties participated in the School Health Index project despite academic pressures and limited resources.

**Methods:**

The Border Health Strategic Initiative supported the hiring and training of an external coordinator in each county who was not part of the school system but who was an employee in an established community-based organization. The coordinators worked with the schools to implement the School Health Index, to develop action plans, and to monitor progress toward these goals.

**Consequences:**

The School Health Index process and school team participation varied from school to school. Individual plans were different but all focused on reducing in-school access to unhealthy foods, identified as high-fat and/or of low nutritional value. Ideas for acting on this focus ranged from changing the content of school lunches to discontinuing the use of nonnutritious foods as classroom rewards. All plans included recommendations that could be implemented immediately as well as those that would require planning and perhaps the formation and assistance of a subcommittee (e.g., for developing or adopting a district-wide health curriculum).

**Interpretation:**

After working with the School Health Index, most schools made at least one immediate change in their school environments. The external coordinator was essential to keeping the School Health Index results and action plans on the agendas of school administrators, especially during periods of staff turnover. Staff turnover, lack of time, and limited resources resulted in few schools achieving longer-term policy changes.

## Background

Adult U.S. Hispanic populations living along the Arizona, United States-Sonora, Mexico border experience type 2 diabetes prevalence rates that are double the rate of the general U.S. population (1,2). The rate of type 2 diabetes is also rising among youth, especially in Mexican American children (3,4). School nurses in the border region report that the number of children with diabetes in their schools is increasing rapidly. Risk factors contributing to these rates are ethnicity, family history, obesity, physical inactivity, and poor nutrition (5).

Increases in rates of diabetes are closely associated with obesity rates. Obesity rates among U.S. children have been escalating rapidly over the past three decades (4,6,7). Data from the 2003 Centers for Disease Control and Prevention (CDC) Youth Risk Behavior Surveillance System show that 14% of Arizona high school students were at risk for becoming overweight, and 11% were overweight. While this was slightly lower than the U.S. estimates of 15% at risk and 14% overweight (8), no data are available for children living along the border. If extrapolations are made from adult data from the region (1,2), more children living along the border are at risk and are overweight than the general U.S. population. To reverse this trend of increasing obesity and diabetes in youth along the U.S.-Mexico border, interventions must target two modifiable risk factors: physical inactivity and poor nutrition.

Schools are ideal environments for promoting physical activity and good nutrition (9,10). Unfortunately, U.S. schools face many barriers to having healthy environments. The reduction or elimination of physical education (PE), the transfer of school food service to outside vendors, and reliance on vending machine revenues for extracurricular activities all contribute to a less-than-optimal health environment for children. In addition, these factors may be contributing to the dramatically increasing rate of childhood obesity in the United States (4). Policies and resources shaping the school environment impact students' patterns and levels of physical activity (9,10) and patterns of food and nutrient intake (11).

To address physical activity and nutrition in the school environment, the CDC developed the School Health Index for Physical Activity and Healthy Eating: A Self-Assessment and Planning Guide (SHI) in 2000 (12)To address physical activity and nutrition in the school environment, the CDC developed the School Health Index for Physical Activity and Healthy Eating: A Self-Assessment and Planning Guide (SHI) in 2000 (12). The SHI enables schools to 1) identify strengths and weaknesses of physical activity and nutrition policies and programs; 2) develop action plans for improving student health; and 3) involve teachers, parents, students, and the community in improving school services. The SHI manual consists of eight modules drawn from the CDC Coordinated School Health Program model. The SHI is a team-based assessment process. Recommended team members include administrators, teachers, school health workers, food service personnel, parents, and community health agencies. Team members respond to a series of questions in each module, and the questions are scored to yield an index reflecting their school's strengths and weaknesses. The SHI also includes a planning section that helps schools use the index scores to develop action plans (12).

Between 2000 and 2003, the SHI was implemented in 13 schools in two Arizona-Sonora border counties as part of the Border Health Strategic Initiative (*Border Health ¡SI!*) (13).* Border Health ¡SI!* was a legislative appropriation for a comprehensive diabetes prevention and control program in Cochise, Santa Cruz, and Yuma, Arizona counties.* Border Health ¡SI!* consisted of policy coalitions and interventions targeting providers, people with diabetes, their families, the general community, and schools in two of the border counties. This paper describes the schools component of *Border Health ¡SI!*. It provides a case study of the SHI implementation process for seven elementary schools and the barriers to change encountered in the school environment.

## Context

Schools were recruited from the Nogales area of Santa Cruz County and the communities of Somerton and San Luis in Yuma County, Arizona. Nogales had a population of approximately 21,000 in 2001 and is predominantly Hispanic (97%) (14). The majority of individuals (64%) had incomes less than 200% of the federal poverty level. Most adults (52%) did not have a high school diploma, and 17% were unemployed (14).

*Border Health ¡SI!* recruited eight schools from three public school districts in the Nogales area. Combined, these districts serve approximately 9256 students and have 10 elementary schools, three middle schools, and three high schools. Of these 16 schools, six did not meet the federal Leave No Child Behind criteria in 2003, and two were underperforming (15-17). No schools were classified as excelling. During the first two years of *Border Health ¡SI!*, one district was on a year-round calendar. In 2002, this district resumed a traditional calendar. A small district (made up of one school) kept the year-round schedule.

In the Yuma area, five schools were recruited from the communities of Somerton and San Luis, which are 100% Hispanic. Combined, the two communities had a population of approximately 24,610 in 2001 (18,19). Between 63% and 78% of the population had incomes less than 200% of the federal poverty level. The majority of adults (62%–65%) did not have a high school diploma, and a large percentage (44%–66%) was unemployed (18,19).

*Border Health ¡SI!* worked with two public school districts in Yuma County. At that time, the districts served approximately 6524 students from K–12, with six elementary schools, two middle schools, and one high school. Of the nine schools, four were ranked by the state of Arizona as underperforming, and four did not meet the federal Leave No Child Behind criteria in 2003 (20,21). No schools were classified as excelling. Because of exploding population growth, two schools in one district were running double sessions (7:00 AM–12:25 PM and 12:30 PM–6:00 PM) with two sets of principals and teachers. This schedule did not allow time for extra activities or even a vacant meeting room.

## Methods

### Selection of SHI

The community-based agencies involved with *Border Health ¡SI!*, along with technical assistance from the Mel and Enid Zuckerman Arizona College of Public Health (MEZACOPH), selected the recently released SHI as a tool that would enable schools to start thinking about creating healthier environments. Despite the lack of published evaluation results, the *Border Health ¡SI!* group felt that it was a reasonable tool to focus schools on physical activity and nutrition policy. The SHI and follow-up were the only school-based interventions as part of *Border Health ¡SI!*.

### Project design

The University of Arizona Cooperative Extension in Yuma County and Southeast Arizona Area Health Education Center (SEAHEC) in Santa Cruz County facilitated implementation of the SHI. These two community-based agencies had strong existing relationships with local schools. SEAHEC was involved in a variety of school nutrition education programs, and Cooperative Extension was responsible for the 4-H clubs for children and thus also worked closely with area schools.

The community agencies and others involved in *Border Health ¡SI!* expressed concern that the schools were overburdened and that health promotion and chronic disease prevention might not be high priorities. We believed, however, that an outside advocate could discuss the serious issues related to chronic disease and how they impact children. We also believed that resource-stressed schools would accept external coordinators from established and trusted agencies to provide assistance and support. Cooperative Extension and SEAHEC identified staff members who could serve as external coordinators to assist schools in completing the SHI assessment and planning process and in coordinating and compiling the SHI materials. MEZACOPH staff provided education to external coordinators on the SHI and the relationship between adolescent health and chronic disease. External coordinators were responsible for documenting recruitment efforts, the SHI process within the schools, team member activities, and action plans.

### School recruitment

External coordinators initiated the recruitment process by presenting the SHI to school district superintendents or assistant superintendents, the school boards, or directly to principals. In addition, external coordinators contacted schools where they had personal connections. By the end of the third year, all schools in the area were approached, and any that expressed interest were contacted. The external coordinator provided a verbal overview and copy of the SHI, and if received positively, made a presentation to school personnel. When schools were hesitant to participate, additional assistance was sought from two directors of health services, a school board president, and a registered nurse at a school-based clinic to encourage schools to participate. Schools were offered a financial incentive of $1500 upon completion of the SHI. The incentive was provided by Cooperative Extension and SEAHEC. Schools were encouraged to apply the funds toward their action plans but were not required to do so. At the end of the three-year period, the SHI was implemented in 10 elementary schools, two middle schools, and one high school, about half of the 25 schools approached.

### Implementation of the SHI

Once a school agreed to complete the SHI, the principals identified an internal SHI coordinator. The internal coordinator recruited team members, and the external coordinator met with the team to provide materials and an overview and outline the assessment process. The external coordinator then followed up with the internal coordinator to ensure that the teams were meeting, collected scorecards, produced a summary document, and scheduled and helped conduct the action planning session.

### Evaluation plan

Because *Border Health ¡SI!* was a legislative appropriation and was not funded as a research project, resources were not available to do an in-depth study of the SHI. However, the community-based agencies and MEZACOPH reached an agreement on an evaluation plan. Documentation of the plan would include information on the interest schools showed in completing the SHI, the ability of SHI teams to understand and complete the SHI, the process for completing the evaluation instrument, whether or not the teams created action plans, and finally, whether or not schools were able to make any changes suggested in the action plans.

Detailed quarterly reports by Cooperative Extension and SEAHEC were used to document school interest and the SHI process. MEZACOPH staff conducted in-depth interviews with the external coordinators and with a convenience sample of 14 SHI team members from the first five schools to complete the SHI. The purpose of the interview was to identify barriers to implementing the SHI, to find out whether or not SHI team members believed an external coordinator was necessary, and to identify changes in the school environment that could be attributed to the process. The external coordinators were in frequent contact with the schools after completion of the SHI. A year after completion of the SHI, external coordinators contacted the schools to determine whether or not SHI action plans were being implemented.

## Consequences

The SHI process was different in each of the schools. The time frame for complete implementation ranged from four weeks to almost two academic years. Most schools completed the SHI within one semester. Schools were encouraged to complete the SHI during the fall semester to allow for time to work on action plans. Unfortunately, all schools completed the SHI during the spring semester.

The SHI team composition varied by school. Team size ranged from six to 34. All schools included at least one parent. As described earlier, the border communities are predominantly Hispanic; therefore, at several schools, parents emphasized the need for a Spanish version of the SHI. A health educator and social worker were not included for Yuma area schools because these positions do not exist in these schools.

An in-depth interview with 14 SHI team members revealed that team members felt that the SHI helped to build awareness of school commitment, identify changes that do not require resources, encourage policy and action, bring health issues to the schools' attention, and raise awareness of federal policies. The team members also identified the four key barriers to implementing the SHI: 1) time, 2) getting people to meetings, 3) initial buy-in, and 4) perceived lack of expertise. The SHI team members believed that the key roles played by the external coordinators were facilitation and guidance. The external coordinators also assisted in overcoming barriers.

No school included representatives from community health agencies on the SHI team. Although outside community members were suggested by SHI guidelines, both internal and external coordinators felt that these individuals did not have the in-depth knowledge of the school environment necessary to answer the detailed SHI questions. The external coordinators frequently filled this role as representatives of the community. As part of the *Border Health ¡SI!*, community coalitions or Special Action Groups (SAGs) were established to focus on policy change to create healthier communities (22). The external coordinators regularly updated these coalitions on SHI progress in schools, and the coaltions served as resources to the schools. Coalition members included the external coordinators, school administrators, nurses, teachers, and a wide variety of community agencies (22).

### Santa Cruz County

By the end of *Border Health ¡SI!*, four elementary schools, one K-8 school, two middle schools, and one high school completed the SHI in Santa Cruz County. During the final quarter of the project period, the director of health services for one district (two elementary schools, one middle school, and one high school) used SHI results to develop the district's comprehensive health plan. Results were not available from the individual schools, and the district health plan is not complete at this writing. The information reported here is based on reports from three of the elementary schools representing two school districts. Overall, half of the schools in the area completed the SHI. The main reasons for schools refusing to participate were lack of time and lack of a school champion. When a school champion was identified, time became less of a barrier.

The intent of the SHI is for SHI teams to collaboratively develop action plans based on the results from the eight SHI modules. In two of the Santa Cruz County schools, the SHI was completed at the end of the semester, and the teams were unable to develop action plans. The principals, who had participated on the teams, created the action plans in isolation. This process was especially problematic because one of the principals resigned shortly after creating a plan. In fact, by the beginning of the next academic year, only two of the seven SHI team members were still at their school (hereafter referred to as Santa Cruz School 1 [SC1]). The second principal to develop action plans promoted many school-level changes (Santa Cruz School 2 [SC2]). Unfortunately, she also left her position.

#### Case Study 1

SC1  made several changes as it implemented the SHI, including the decision to move all candy sales out of the cafeteria. Most notably, the school hired a full-time PE teacher and developed a policy that prohibited the selling of junk food for fundraisers. The fundraising policy came into question the following academic year when the loss of almost all members of the SHI resulted in very little institutional memory. This case offers an example of how important the external coordinator's role is in the process. The remaining SHI team member was uncomfortable promoting the action plans to the new principal. The external coordinator contacted the principal early in the school year to discuss the SHI and action plans. The long-term goal of the SHI team was to increase the length of the lunch period. The principal was unfamiliar with the SHI but expressed verbal support for the action plans. When resistance developed over the fundraising policy, the principal enlisted the aid of a remaining SHI team member to explain the policy to the staff and parent-teacher organization. The policy was upheld. SC1 had a new principal the following year. The external coordinator continued to follow up and provided the impetus for the principal to create a new SHI team that completed the SHI a second time. SC1 has had a full-time PE teacher for three years. They have not lengthened the school lunch period. Fundraisers now include gifts, wrapping paper, and magazines as well as chocolate. Items for sale at lunch include pencils, notebooks, pickles, oranges, peanut butter crackers, and *salditos* (salted dried prunes) instead of baked goods and junk food.

#### Case Study 2

The principal/superintendent at SC2 was deeply committed to the SHI process. The school made several immediate changes. The school bake sales were converted to healthy snack sales. Graduation cookies and punch were cancelled and replaced with lemonade and baked chips and salsa. As a result of the SHI, the school hired a full-time PE teacher and developed a PE course. The goal was to have a letter grade for the course, not a pass/fail grade. An existing staff member was certified as a PE instructor. The school also organized track and basketball teams for the first time. The following summer, the school board attempted to drop the program. The principal called the *Border Health ¡SI!* coalition for assistance. Coalition members, teachers, and parents attended the school board meeting. The principal presented the SHI results, and the teachers and parents strongly supported the program. The PE program was not cut. Unfortunately, before the next academic year, the principal accepted a job as a district superintendent in another community. In that role, she was able to add PE into all elementary schools. The PE course is pass/fail. Individual teachers are offering low-fat snacks and less sweet snacks at parties, but this is not a coordinated school effort. Carrots, orange juice, milk, graham crackers, and cheese sandwiches are served at the school open house.

#### Case Study 3

The third Santa Cruz school (SC3) to complete the SHI took one and a half years to complete the process. The school was undergoing major renovations when it was first contacted and despite expressing interest, it was unable to commit to the process. Once the school committed, however, the external coordinator reported that the SC3 SHI team was the most enthusiastic. The school removed the vending machine from the cafeteria and decided to remove all other soda and candy vending machines and replace them with healthier choices. The beverage machines include fruit juices, water, tea, and lemonade. The SHI team also recommended removing the school store from the cafeteria. The SHI team reported their results to the *Border Health ¡SI!* coalition. The team was encouraged by the coalition to write a newspaper article for the local paper describing the changes they were making. Students were interviewed by a local television station when the soda was replaced by fruit drinks. The students said they missed the soda "but juice was okay."

The school store has not moved, but it is no longer selling candy. It replaced candy with healthier options, and profits have stayed the same. Bake sales now have oranges, cucumbers, and carrots. Some sales include candy but only small candy bars.

### Yuma County

A total of five schools, four elementary schools and one middle school, implemented the SHI as part of the *Border Health ¡SI!* in the southern Yuma area. All three elementary schools in one district participated. In the second district, only one elementary school participated. It was the only elementary school not running double shifts. Overall, five of the nine schools in the area completed the SHI. The main reason for refusing to participate was lack of time. In Yuma, the external coordinators actively facilitated the action planning process. Documentation from the Yuma area focuses on the action plans.

#### Case Study 1

All teachers participated on the SHI teams at the first school (YC1) in the Yuma area. Not all teachers were enthusiastic. A school policy was developed to prohibit the use of food as a reward in the classroom, and students were limited to one snack per day. The SHI team also set goals of 1) teaching more nutrition information, 2) gathering and disseminating information on all food and drink items sold at the school, and 3) shifting from selling junk food items and sports drinks to healthier options. The school offered granola and fruit bars as alternatives, but they did not sell, and the school reverted to selling junk food. YC1 established walking groups two to three days per week for students. Teachers organized structured fitness breaks. The school also implemented an annual field day for staff and students.

#### Case Study 2

YC2 also established a school policy prohibiting nonnutritious food as a reward and limiting snacks to one per day per student. The SHI team was especially interested in eliminating outdated health education material and incorporating a sequential health education curriculum. Teachers began standing at the salad bar to encourage children to eat more fruit and vegetables. The school also began to increase use of community facilities and to offer a swimming class at the local community pool. The school's part-time PE teacher left the school for a full-time position out of the district. The SHI team presented the SHI results at a staff workshop.

#### Case Study 3

YC3 established a lengthy list of goals, including requiring hand washing, adopting a sequential health education curriculum, and incorporating activity breaks into the classroom. Two years after implementation of the SHI, no progress has been made.

The three Yuma area elementary schools described here are from one school district. The superintendent and SHI team members requested that the external coordinators from Cooperative Extension present the SHI results to the school board. The perception was that the board would view the external coordinators as community members and not as school personnel, and they would thus have a greater impact. The SHI team members then presented the results to the *Border Health ¡SI!* coalition.

#### Case Study 4

The fourth Yuma area elementary school (YC4) was from the second school district and focused on similar issues as the other three schools. This school chose to focus on increasing instruction time for health and PE, adopting new health education textbooks, and presenting health-related information to parents and staff. At this point, no progress has been made toward these goals.

The action plans of these schools emphasized modifying health-related curricula and adopting a sequential health education curriculum. Upon exploring the issue at the district level, it was determined that the district is not considering modifications to health education curriculum until 2005. To provide the teachers with resources, Cooperative Extension presented a resource event where teachers selected educational materials focusing on health education, nutrition, and physical activity and provided suggestions on ways to incorporate nutrition messages and physical activity into the existing curricula. In addition, Cooperative Extension also provided one school library with more than 50 books on physical activity and nutrition (fiction and nonfiction).

## Interpretation

Seven Arizona elementary schools along the Arizona-Sonora border assessed their school environments and developed action plans using the CDC's SHI. Despite some difficulties resulting from time constraints and human resources, all schools were able to complete the assessment. The schools focused their action plan priorities on nutrition and physical activity. School action plans included items that could be addressed immediately (e.g., a school policy prohibiting use of candy as rewards) as well as policies that would necessitate implementation at the district level (e.g., adopting a sequential health curriculum or seeking funds to hire a full-time PE instructor). The individual school action plans varied, but all seven shared one component: to reduce internal access to unhealthy foods. Plans included changing the content of school lunches, discontinuing the use of nonnutritious foods as classroom rewards, moving candy sales and a snack bar away from the cafeteria, and choosing healthy alternative fundraisers. Two schools were able to use SHI results to hire PE teachers.

The goal of *Border Health ¡SI!* was for schools to disseminate the results of the SHI at least to the teachers at each participating school. In addition, we hoped that the results would be presented at parent-teacher and school board meetings. High rates of staff turnover highlight the importance of disseminating SHI results and action plans to a larger audience. It is crucial for schools to have an advocate for physical activity and nutrition. The external coordinator and *Border Health ¡SI!* coalitions served as those advocates and were able to keep the issue of school health prominent in school administrators' minds.

The number of underperforming schools in these districts and the lack of highly performing or excelling schools results in stress on the educational system. Principals and school board members are under pressure to improve the academic performance of their schools. When approached for funds or time to implement new programs, school administrators frequently cite the need for scholastic improvement above all other issues. External coordinators returned repeatedly to meet with school administrators to discuss the importance of physical activity and good nutrition. An additional tactic for educating administrators was to recruit them to participate in the *Border Health ¡SI!* SAGs.

The current educational system is responsible for a wide variety of activities: academic performance, social services, childhood immunizations, and the nutrition of students through the federal school breakfast and lunch programs. Involving schools in health promotion and disease prevention activities is critical, but we must recognize that most school systems are under extreme pressure to demonstrate academic progress. The fact that half of schools approached in two low socioeconomic areas participated in the SHI process indicates the commitment of school personnel to the overall health and well-being of their students and communities. The support of an external coordinator from a local agency can assist in removing some logistical barriers.

While most schools were extremely committed and enthusiastic about SHI action plans, staff turnover, time, and limited resources were barriers to progress even with the support of an external facilitator. Implementation of new programs is limited further by the low number of certified PE and health education specialists employed by the districts. Change in staff in one district occurred during the first year, and staff had to be educated about the SHI action plans and school goals. Some schools were undergoing major renovations and building projects, and in some cases, new schools were built. These schools found it challenging to take on new projects like the SHI.

One cautionary note is that publicity may present a barrier to acceptance of the SHI process. In the opinion of the external coordinators, publicity surrounding the removal of soda machines at one school may have discouraged other schools from participating in the SHI process. When funding for schools is so tight that vending machine sales are used to support photocopying expenses, field trips, graduation ceremonies, and extracurricular events, the threat of losing those funds can deter schools from eliminating this source of revenue, even though they are interested in improving the health of their students. The SHI and our project did not push schools to remove vending machines; instead, we encouraged schools to identify the best priorities for their own schools. These messages were lost by the news media. The external coordinator sought advice from the *Border Health ¡SI!* SAGs on how to convey our messages. The negative impressions receded after a brief time, and five more schools completed the SHI by the end of *Border Health ¡SI!*.

This project showed the value of having an external coordinator to help with continuity and with keeping the project top-of-mind with school officials, especially during periods of high staff turnover. In addition, the external coordinators acted as a resource beyond coordination. External coordinators created resource manuals for alternative ideas for school fundraisers, linked schools with other community resources, found funds to provide teachers with educational materials, and volunteered at school events. Although the SHI process does not include incentives, we found that monetary incentives for carrying out policy priorities seemed to encourage participation and gave schools resources to implement policies. Overall, most schools were able to implement immediate changes. Policies requiring a longer-term process and additional resources were more difficult to carry out. Future projects should focus on documenting whether students increased their physical activity or improved their eating habits as a result of SHI policies.
